# Effect of a Natural Processing Aid on the Properties of Acrylonitrile-Butadiene Rubber: Study on Soybean Oil Fatty Acid from Seed Crop

**DOI:** 10.3390/polym13203459

**Published:** 2021-10-09

**Authors:** Phattarawadee Nun-Anan, Chesidi Hayichelaeh, Kanoktip Boonkerd

**Affiliations:** 1 Center of Excellence on Petrochemical and Materials Technology, Bangkok 10330, Thailand; phattarawadee.anan@gmail.com; 2 Department of Materials Science, Faculty of Science, Chulalongkorn University, Bangkok 10330, Thailand; h.chesidi@gmail.com; 3 Green Materials for Industrial Application Research Unit, Faculty of Science, Chulalongkorn University, Bangkok 10330, Thailand

**Keywords:** vegetable oil, processing aid, soybean oil fatty acid, acrylonitrile-butadiene rubber, vulcanizate

## Abstract

For the industrial production of rubber, one of the key ingredients is a processing aid. It not only facilitates the processability but also tunes the final properties of the resultant rubber. In general, for a polar rubber like acrylonitrile-butadiene rubber (NBR), the processing aids earning the most attention are synthesized from petroleum, such as dioctyl phthalate (DOP). However, due to their toxicity, many rubber chemists have tried to find alternative chemicals that are environmentally friendly and derived from a renewable resource. In this research, we investigated the effects of the soybean oil fatty acid (SBOFA), synthesized in house via hydrolysis of SBO, on the properties of NBR in comparison with DOP. Initially, it was found that the addition of SBOFA improved the flowability of the NBR compound, as indicated by the progressive decrease in the Mooney viscosity with increasing levels of SBOFA. The results from various techniques indicated that the crosslink density of the NBR vulcanizates passed through the maximum at the SBOFA loading of 4 phr. Upon loading SBOFA up to 4 phr, there was no significant deterioration in the mechanical strength of the SBOFA-plasticized NBR vulcanizates. Typically, the presence of SBOFA at 4 phr enhanced the thermal resistance of the NBR vulcanizate by shifting the thermal decomposition to a higher temperature. At a given loading, it was found that the SBOFA-plasticized NBR vulcanizate showed a comparable plasticizing efficiency and mechanical strength with the DOP-plasticized one. The result from this study shows that SBOFA is a good alternative sustainable eco-friendly processing aid to use for NBR.

## 1. Introduction

Processing aids are one of the major nonvolatile additives in the rubber industry. Typically, a processing aid is added to rubber compounds as a softener to improve the processability (reduced viscosity) and, in particular, to improve the filler dispersion in the case of high filler loading or the other ingredients during rubber processing [1,2].

Over the past decade, petroleum-based oils, including aromatic, paraffinic, and naphthenic oils, have become common processing aids, but they are nonrenewable and indestructible materials. They have mostly been used as a processing aid in the rubber industry, especially in tire products, because their price is lower compared to other types of processing aids and they have a good balance of properties [3]. However, in 1994, the Swedish National Chemical Inspectorate described the hazard of using aromatic oils and distillate aromatic extracts (DAEs) as extender oils in tire rubber, due to their high content of polycyclic aromatic (PCA) compounds that are harmful to health, safety, and the environment [4,5]. Meanwhile, the polycyclic aromatic hydrocarbon (PAH) content in aromatic oils has been shown to be harmful to humans and the environment, according to the regulation (EC) No 1907/2006 of the European Parliament and of the Council Regulation Concerning the REACH legislation [6]. Thus, although these petroleum-based oils are good extender oils in both nonpolar (i.e., natural rubber (NR) and styrene butadiene rubber (SBR)) and polar elastomers (acrylonitrile-butadiene rubber (NBR)), due to their polarity that improves the compatibility with elastomers [5,7,8], they are classified as carcinogens. As a result, there has been intensive research to develop eco-friendly rubber processing aids to reduce the consumption of nonbiodegraded oils (or petroleum oil) in the rubber industry, while still maintaining the plasticizing efficiency while reducing the toxicity of the processing aid.

Vegetable oils (i.e., soybean, palm, castor, and sunflower oils), are renewable oils that are derived from natural plant sources and can be found in all regions of the world [9]. The specific characteristics of these vegetable oils include that they are sustainable, safe, environmentally friendly, biodegradable, and, especially, less costly [8]. According to these advantages they appear as potential substitutes to petroleum derivatives and their role as a reliable material within rubber processing is currently being considered as a first priority in the rubber industry.

Extensive research is ongoing to apply vegetable oils as a processing aid in both nonpolar and polar elastomers. Various effects of vegetable oil on the properties of rubber compounds and their vulcanizates (i.e., Mooney viscosity, cure characteristic, mechanical, and dynamic properties) have been evaluated [10,11,12,13,14,15]. In nonpolar elastomers, it has been reported that castor and jatropha oils can effectively replace aromatic oils in SBR, because they give SBR vulcanizates an improved abrasion resistance and rebound resilience [10]. It was also reported that the use of soybean oil (SBO), as well as sunflower and palm oils, as a processing aid could improve the dispersion of carbon black (CB) in the rubber matrix compared to the one without a processing aid [11]. The advantages of using SBO and polymerized SBO as processing aids in NR/SBR vulcanizates were reported to be a greater flowability together with better mechanical and dynamic properties when used to replace naphthenic oil [12].

In addition, previous studies have established that vegetable oils, including SBO and sunflower and palm oils, can be utilized as processing aids and suitable replacements for petroleum-based oils (i.e., aromatic and paraffinic oils) in nonpolar rubbers [11,13]. Hence, some studies have attempted to use vegetable oils in polar elastomers. The influence of rice bran oil (RBO) and the use of commercial dioctyl phthalate (DOP) oil as processing aids in polar elastomers (i.e., NBR and chloroprene rubber (CR)) was also compared [14]. It was found that RBO can function as a multipurpose (coactivator and antioxidant) additive in NBR and CR, and can be applied as a processing aid to replace DOP [14]. Furthermore, comparison of the performance of maleated castor oil (MACO) and DOP on NR, NBR, and ethylene propylene diene monomer (EPDM) rubbers revealed that the MACO as a processing aid in NR, NBR, and EPDM rubbers gave a similar flowability to the DOP. In addition, MACO had a longer cure time with a similar scorch safety as DOP [15]. Thus, vegetable oils are extensively utilized as a replacement for petroleum-based oils in the rubber industry.

The outcome of this study is to provide an optional processing aid derived from a renewable resource with at least comparable efficiency to a commercial processing aid. This will offer an environmentally friendly rubber compound and simultaneously reduce the carbon footprint generated by the production of petroleum-based processing aids. Among the naturally present vegetable oils, SBO is the most cultivated oil seed in the world. Global soybean production accounts for approximately 56% of global oil seed production [9], with year-end stocks ranging from 34–52 million tons from 2014–2019 [16]. In addition, the large production and low cost of SBO makes it a potentially alternative natural oil that could fill the requirements in the rubber industry of a good processing aid [14]. However, most of the studies to date have not specified the advantages of using soy bean oil fatty acid (SBOFA) derived from SBO as a processing aid in polar elastomers (such as NBR). Therefore, the aim of this study is to investigate the effect of different SBOFA loadings (i.e., 0, 2, 4, 6, and 8 phr) on the cure characteristic, crosslink density, mechanical properties (i.e., modulus, tensile strength, elongation at break, and hardness), thermal resistance, and thermo-mechanical property of NBR vulcanizates and also to compare the efficacy of SBOFA when used as a processing aid with that of the commercial processing aid, dioctyl phthalate (DOP).

## 2. Materials and Methods

### 2.1. Materials

The chemicals used in this work are summarized in Table 1.

### 2.2. Preparation of SBOFA

The SBOFA was prepared by saponification of SBO as previously described [17]. In brief, the SBO was first hydrolyzed by sodium hydroxide (NaOH) at 70 °C for 9 h at a 1:6 molar ratio of oil: NaOH. Then, the mixture was transferred into a separating funnel, set for 1 h, and then washed with 20% saturated aqueous sodium chloride (NaCl) at room temperature (27 °C). The resulting two aqueous layers, which contain glycerin and sodium sulfate, were completely separated. The glycerin phase (upper layer) was further leached with hydrochloric acid (HCl) for acidification. Finally, the organic phase (SBOFA) was leached with warm (about 60 °C) distilled water to neutralize it. The water contaminating the neutralized organics phase was removed using a rotary evaporator (IKA Inc., Wilmington, NC, USA).

### 2.3. Characterization of SBO and SBOFA

The structure of the SBO and corresponding SBOFA was analyzed using an attenuated total reflectance (ATR)-FTIR analysis (Nicolet^TM^ iS50 FTIR Spectrometer, Thermo Fisher Scientific Inc., Waltham, MA, USA). Each measurement was performed for 32 scans over a wavelength range of 4000–400 cm^−1^ with a resolution of 4 cm^−1^. All ATR-FTIR spectra were normalized.

### 2.4. Preparation of Rubber Compounds

Mixing of the NBR compounds was performed by a two-stage method with the formulations of the rubber compounds summarized in Table 2. The master batch (compound without curing agent) was first prepared using an internal mixer (Charoen Tut Co., Ltd., Bang Phli, Samut Prakan, Thailand) with a fill factor of 0.8. The starting temperature of the mixer was set at 60 °C, with a rotation speed of 60 rpm. The NBR was mixed for 2 min and then the respective processing aid (SBOFA or DOP) was added and continuously mixed for a further 4 min. Next, the master batch was dumped and immediately sheeted out using a laboratory two-roll mill (Labtech Engineering Co., Ltd., Mueang Samut Prakan, Samut Prakan, Thailand). The sheeted master batch was left to cool to room temperature.

The second mixing stage was performed using a two-roll mill. The initial mixing condition was set at 60 °C with a friction ratio of 1:1.4. The master batch was passed through the gap between the two roll mills and then banded on the front roll. Next, the curing agent (DCP) was added and further mixed with the rubber for 3 min. Finally, the rubber compounds were sheeted out and left at an ambient temperature, before further vulcanizing and testing for the various properties of the NBR vulcanizates.

### 2.5. Cure Characteristics of Rubber Compounds

Cure characteristics of the NBR compounds at 160 °C were investigated using a Moving Die Rheometer (Rheo Tech MD+, TechPro, Piqua, OH, USA). The minimum torque (M_L_), maximum torque (M_H_), torque difference (M_H_-M_L_), scorch (t_s2_), and cure times (t_90_) were recorded.

### 2.6. Preparation of Rubber Vulcanizates

According to the results obtained from the cure rheometer, the NBR vulcanizate was compression-molded at 160 °C for the optimum curing time (t_90_), giving a rubber sheet with dimensions of 120 × 120 × 2 mm^3^.

### 2.7. Characterization

#### 2.7.1. Mooney Viscosity of Rubber Compounds

Mooney viscosity of the NBR compounds was tested according to ISO 289-1, using a MonTech Mooney viscometer (Montech Co. Ltd., Shinjuku, Tokyo, Japan). Each measurement was conducted at 100 °C using a large (L) rotor. Torque (in Mooney unit) was continuously recorded as a function of the time. Mooney viscosity (ML 1 + 4@100) of the rubber compound was the value of torque after preheating for 1 min and test running for 4 min.

#### 2.7.2. ATR-FTIR Spectrum of Unvulcanized and Peroxide-Vulcanized Rubbers

Chemical structures of the unvulcanized and peroxide-vulcanized NBRs were analyzed using an attenuated total reflectance (ATR)-FTIR analysis (Nicolet^TM^ iS50 FTIR Spectrometer, Thermo Fisher Scientific Inc., Waltham, MA, USA). Each measurement was performed for 32 scans over a wavelength range of 4000–400 cm^−1^ with a resolution of 4 cm^−1^.

#### 2.7.3. Crosslink Density of Rubber Vulcanizates

The crosslink density of the NBR vulcanizates was investigated using a swelling test. The rectangular test specimens with dimensions of about 10 × 10 × 2 mm^3^ were weighed, immersed in toluene, and left at an ambient condition for 7 days in the dark. After 7 days, the swollen specimens were removed, quickly blotted with filter paper, and weighed (w_s_). The specimens were then dried in a hot air oven at 60 °C for 24 h and weighed (w_d_). The volume fraction of rubber in the swollen network was determined from Equation (1):

(1) $$\phi_{\rho }=1/\left[\left(1+\left(\frac{w_{s}-w_{d}}{w_{d}}\right)\right)\cdot \left(\frac{\rho_{r}}{\rho_{s}}\right)\right]$$

where $w_{s}$ is the weight of the swollen sample, $w_{d}$ is the weight after drying the sample, $\rho_{r}$ is the density of the rubber, and $\rho_{s}$ is the density of the solvent [18]. Then, the apparent crosslink density of the vulcanizates was calculated using the Flory–Rehner equation [19], as shown in Equation (2):

(2) $$v=\frac{-\left(\ln\left(1-\phi_{\rho }\right)+\phi_{\rho }+\chi \phi_{\rho }^{2}\right)}{V_{1}\left(\phi_{\rho }^{\frac{1}{3}}-\phi_{\frac{\rho }{2}}\right)}$$

where *υ* is the crosslink density (mol/m^3^), $\phi_{\rho }$ is the volume fraction of rubber in the swollen network, $V_{1}$ refers to the molar volume of toluene, and χ is the Flory–Huggins interaction parameter between toluene and rubber, for which the value 0.4 was used according to prior literature [20].

#### 2.7.4. Tensile Property of Rubber Vulcanizates

The NBR vulcanized sheet was cut into dumbbell-shaped specimens according to ISO 37. Tensile test was performed using a universal tensile testing machine (Tinius Olse model 10ST tensile testing machine, Salford, Surrey, UK) with a crosshead speed of 500 mm/min. The mechanical properties, in terms of the 100%, 200%, and 300% modulus together with the tensile strength and elongation at break, were recorded. Note that for each sample, all tensile properties were averaged from five specimens.

#### 2.7.5. Thermal Aging Property of Rubber Vulcanizates

The thermal aging properties of the NBR vulcanizates were performed according to ISO 188 Method A at 100 °C for 48 h, using a circulating hot air oven (Memmert GmbH + Co. KG, Schwabach, Germany). After the aging period, the specimens were cooled to room temperature and rested for at least 24 h before tensile testing. The tensile properties of the aged specimens were also tested in accordance with ISO 37. The percentage of retention in the tensile properties was calculated [21] using Equation (3):

(3) $$\mathrm{Retention}\;\mathrm{in}\;\mathrm{property}\;(\%)=\left(\frac{X_{a}}{X_{0}}\right)\times 100$$

where *X_0_* and *X_a_* are the value of the property before and after aging, respectively.

Furthermore, the thermal aging resistance of the NBR vulcanizates was also indicated here by the aging coefficient (*A_f_*) calculated from tensile strength (TS) and elongation at break (EB) before and after thermal aging [22] using Equation (4):

(4) $$A_{f}=\frac{{\left(\mathrm{TS}\;\cdot \mathrm{EB}\right)}_{\mathrm{after}\;\mathrm{aging}}}{{\left(\mathrm{TS}\cdot \mathrm{EB}\right)}_{\mathrm{before}\;\mathrm{aging}}}$$

#### 2.7.6. Hardness of Rubber Vulcanizates

The hardness of the NBR vulcanizates was measured using a manual Shore A durometer (Shore Instrument & MFG. co, Ltd., Jamaica, NY, USA), according to ISO 7691-1. The test was done at least three times.

#### 2.7.7. Temperature Scanning Stress Relaxation (TSSR) of Rubber Vulcanizates

The TSSR test was used to investigate the thermo-elastic properties and relaxation behaviors of the rubber vulcanizates using a TSSR tester (Brabender, Duisburg, Germany). The dumbbell-shaped specimens were first cut from the NBR vulcanized sheets using a die type 5A, according to ISO 527. The TSSR test of the rubber specimens was performed in an electrically heated test chamber and stretched at a constant strain of 50%. For the first step, the specimen was stretched at a constant strain (i.e., about 50%) at an initial temperature (*T*_0_) of 23 °C for 2 h, to allow the short time relaxation to decay. Then, for the second step, the nonisothermal TSSR test, the test temperature was increased from 23 °C to 220 °C at 2 °C/min. The TSSR test was continued until the stress relaxation was completed or the sample was ruptured.

During the nonisothermal relaxation of the TSSR test, the temperature was continuously raised at a constant rate (*ν*). The resulting nonisothermal relaxation modulus *E*(*T*)*_non_*_-*iso*_ varies during the temperature scan at the constant heating rate (*ν*) and is seemingly a function of the temperature. By differentiating *E*(*T*)*_non_*_-*iso*_ with respect to the temperature *T*, the relaxation spectrum *H*(*T*) is obtained according to Equation (5) [23,24], as shown below:

(5) $$H(T)=-T\;{\left(\frac{dE\left(T\right)}{dT}\right)}_{v=const}$$

#### 2.7.8. Thermogravimetric Analysis (TGA) of Rubber Vulcanizates

The thermal decomposition of the NBR vulcanizates was analyzed using a Perkin Elmer Pyris-7 thermogravimeter (Perkin Elmer, Shelton, CT, USA). The samples were heated from 30 °C to 800 °C at 20 °C/min under a nitrogen gas atmosphere.

## 3. Results and Discussion

### 3.1. Chemical Structure of the Obtained SBOFA

**Figure 1:**
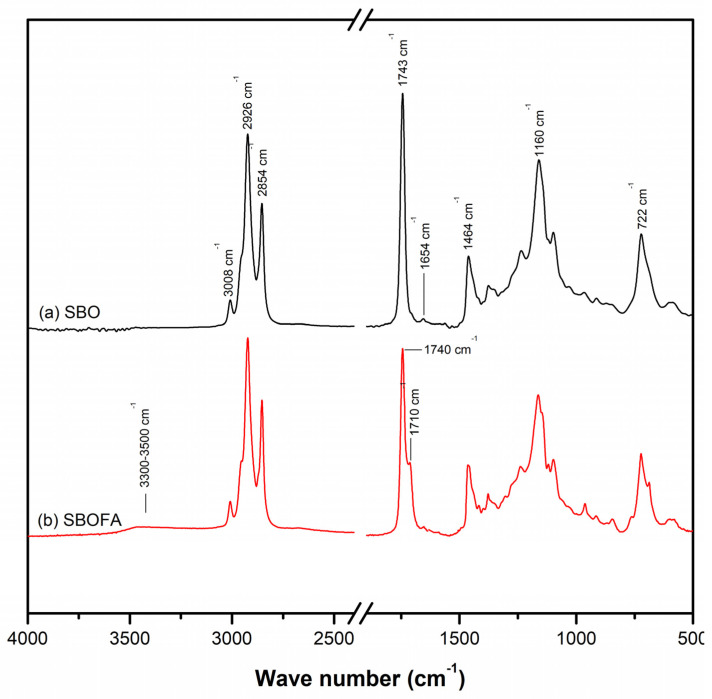
Representative ATR-FTIR spectra of the (**a**) SBO and (**b**) SBOFA.

The SBOFA used as a processing aid for the NBR compound in this study was obtained by alkaline hydrolysis of SBO. To confirm that the SBOFA was successfully obtained, the chemical structure of the SBOFA was investigated using ATR-FTIR analysis in comparison with that of the SBO (Figure 1). The typical functional groups of the SBO and SBOFA appeared at 3008, 2926, 2854, 1743, 1464, 1160, and 722 cm^−1^ and were identified as the –C=CH stretching, C–H of CH_3_ stretching, C–H of CH_2_ stretching, –C=O stretching of ester, C–H of CH_2_, –CH in plane, and –HC=CH– bending (out of plane) [25,26], respectively, as summarized in Table 3.

**Figure 2:**
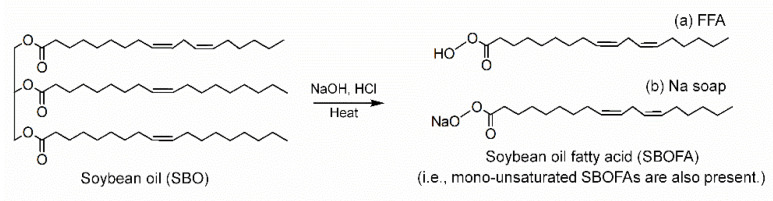
Hydrolysis reaction of SBO and its hydrolyzed products.

From Figure 1, the appearance of peaks at 1710 and 3300–3500 cm^−1^ attributed to the C=O stretching and O–H of the carboxylic acid, respectively, indicated the formation of free fatty acids (FFAs) [25,26,27]. Moreover, the absorption peak at 1743 cm^−1^ of the C=O of triacylglycerols in the SBO was shifted to 1740 cm^−1^ attributed to the carboxylate group of the fatty acid metallic salt (or Na soaps) [27]. Therefore, it was assumed that the alkaline hydrolysis converted SBO to a mixture of FFAs and their sodium salts called SBOFA. The structure of the resultant products obtained after hydrolyzing SBO with NaOH is schematically proposed in Figure 2.

### 3.2. Effect of the SBOFA Loading on the Mooney Viscosity of NBR Compounds

**Figure 3:**
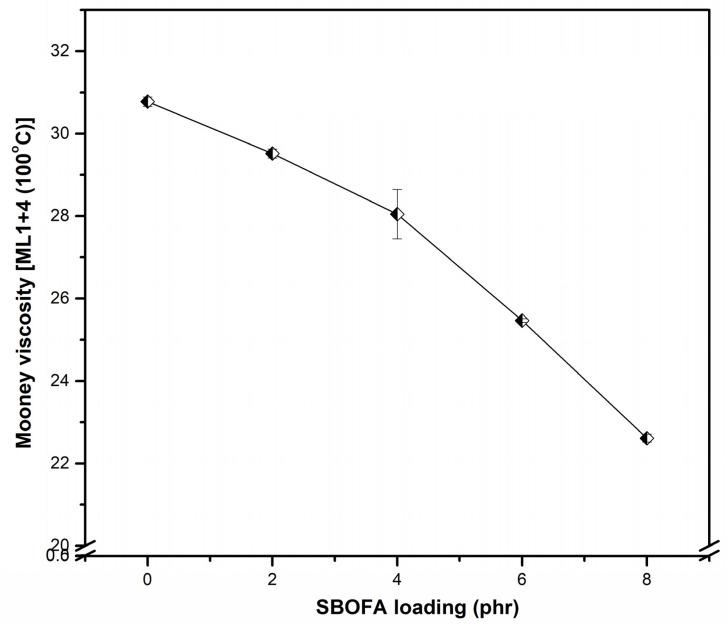
Mooney viscosity of NBR compounds with varying SBOFA loadings. Data are shown as the mean ± 1SD, derived from three experiments.

**Figure 4:**
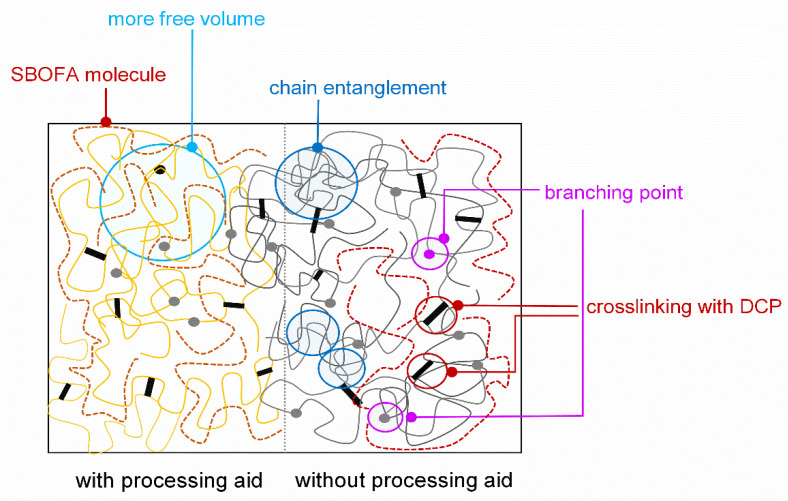
Proposed model for the plasticization with the SBOFA resulting in increasing free volume and chain mobility of the NBR compound.

As mentioned, the aim of this study was to prepare SBOFA and use it as a processing aid for the polar rubber NBR. Therefore, to confirm the efficacy of SBOFA, the Mooney viscosity of the NBR compounds with different SBOFA loadings are shown in Figure 3. Note that the Mooney viscosity reflects the flow behavior and processability of raw rubber and its corresponding rubber compound [2]. The viscosity of the NBR compounds decreased with increasing SBOFA loadings, with the NBR compound loaded with 8 phr SBOFA having the lowest viscosity. Generally, the decreased viscosity indicates the enhanced flowability and could be due to the presence of the long carbon chains that reduce the rubber–rubber interaction or loosen both the physical and chemical crosslinking points, and thus results in an increased mobility of the rubber molecules [13]. Therefore, the higher the SBOFA loading, the lower the friction between rubber chains (lower viscosity) and the more free volume (increased chain mobility) in the NBR compound than in that without the processing aid [17], as schematically proposed in Figure 4.

### 3.3. ATR-FTIR Spectra of the Unvulcanized and Peroxide-Vulcanized NBR without SBOFA

**Figure 5:**
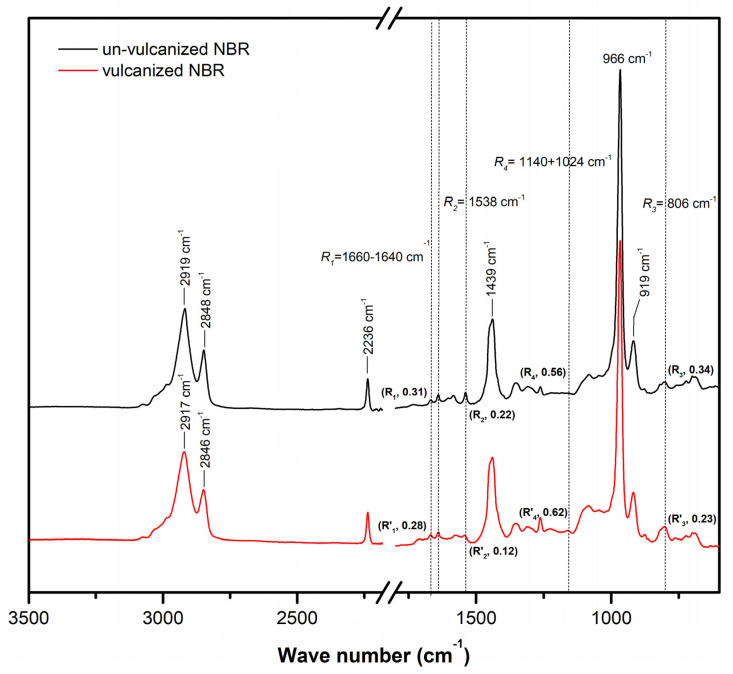
Representative ATR-FTIR spectra of the unvulcanized and peroxide-vulcanized NBR.

Figure 5 compares the ATR-FTIR spectra of the unvulcanized and peroxide-vulcanized NBR. The peak assignment for the infrared spectra is given in Table 3 [28,29]. A clear difference in the unsaturated –C=C– content was observed. The intensity ratios of the peaks at 1660–1640, 1538, and 806 cm^−1^ attributed to C=C stretching to the reference peak at 1443 cm^−1^ attributed to C–H bending in –CH_2_ [28,29] are represented by R_1_, R_2_, and R_3_. As seen after peroxide vulcanization, R_1_, R_2_, and R_3_ decreased. This indicated the reduction of double bond content in the NBR chains [28], thus confirming the occurrence of crosslink reaction. The intensity ratio of the peak at 1140 and 1024 cm^−1^ attributed to C–C stretching to the reference peak at 1443 cm^−1^ attributed to C–H bending in -CH_2_ [30] is represented by R_4_. The R_4_ of the peroxide-vulcanized NBR was higher than that of the unvulcanized one. This confirmed the formation of a new C–C bond (peroxide crosslinking) during vulcanization [30].

**Figure 6:**
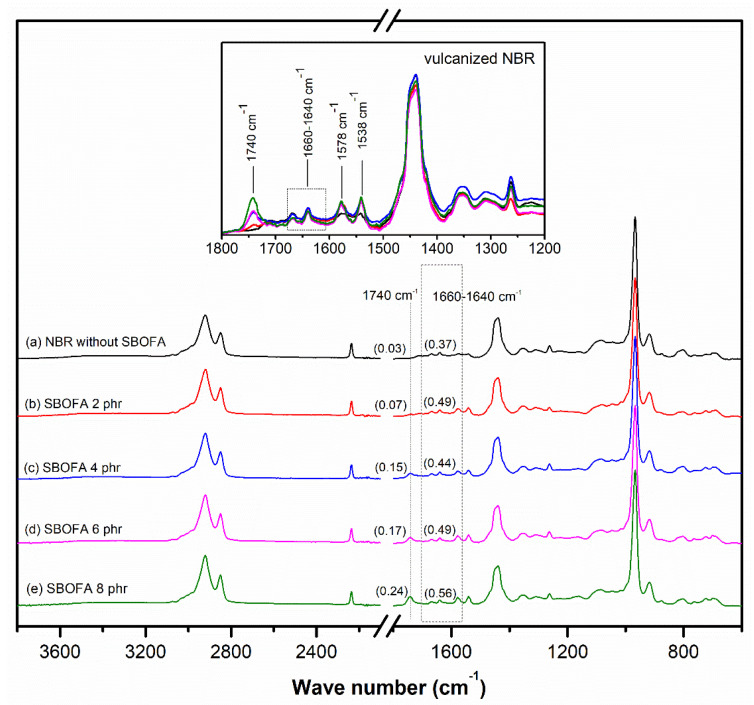
Representative ATR-FTIR spectra of NBR vulcanizates with varying SBOFA loadings.

The ATR-FTIR spectra of NBR vulcanizates with varying SBOFA loadings is shown in Figure 6. All the NBR vulcanizates with SBOFA showed an absorption peak at 1740 cm^−1^ (C=O stretching of the sodium salt fatty acids) [25,31], which was absent in the NBR vulcanizate without SBOFA. Hence, this peak indicated the presence the SBOFA. In addition, the intensity ratio of the peak at 1740 cm^−1^ to the reference peak at 1443 cm^−1^ increased with increasing SBOFA loading levels due to the greater amount of esterified functional groups with increasing SBOFA loading (Figure 6 and Table 4). In addition, new peaks at 1538 cm^−1^ and 1578 cm^−1^ appeared in all NBR vulcanizates with SBOFA after vulcanization, which were attributed to the asymmetric and symmetric coupled C–O stretching of carboxylate anions [27,30]. Furthermore, it was seen that the NBR vulcanizates with varying SBOFA loadings showed the difference in the intensity ratio of the peak at 1660–1640 cm^−1^ (attributed to the double bond content) to the reference peak at 1443 cm^−1^. This change in the double bond contents may not only correlate to the double bonds of rubber molecules but also to the concentration of unsaturated fatty acids in the SBOFA. Hence, the change in the double bond content of NBR molecules may affect various properties of the NBR vulcanizates. Note that the rubber vulcanizate with SBOFA at 4 phr gave the lowest intensity ratio. This will be described in a later section.

### 3.4. Effect of the SBOFA Loading on the Cure Characteristics of NBR Compounds

**Figure 7:**
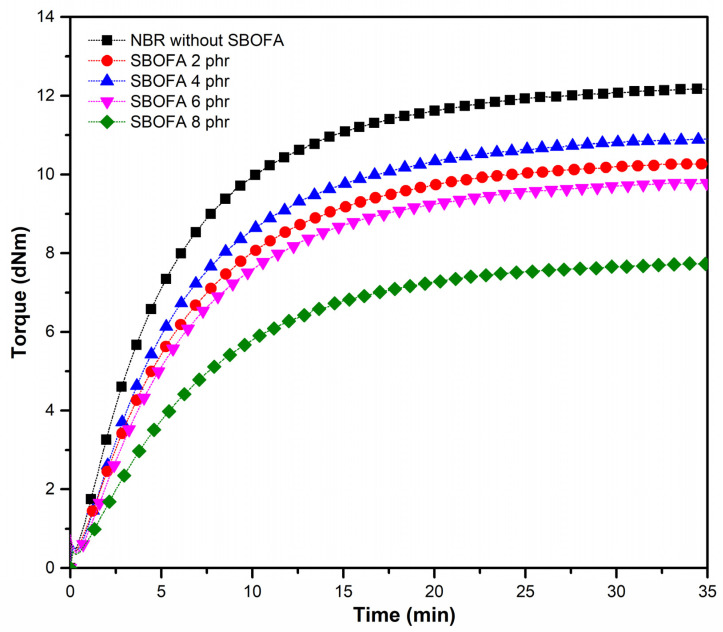
Cure curves of NBR compounds with varying SBOFA loadings.

Figure 7 and Table 5 show the cure characteristics of NBR compounds with varying SBOFA loadings (0–8 phr). The presence of SBOFA significantly reduced the maximum torque (M_H_) and torque difference (M_H_-M_L_) values of the NBR compound compared to that without SBOFA. This is due to the reduction of the rubber–rubber interaction and increased free volume, as previously mentioned.

**Figure 8:**
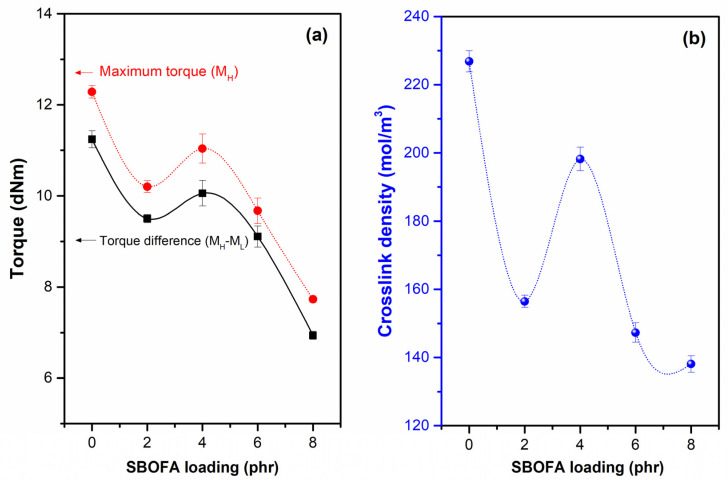
The (**a**) maximum torque (M_H_) and torque difference (M_H_-M_L_) and (**b**) crosslink density of NBR compounds with varying SBOFA loadings. Data are shown as the mean ± 1 SD, derived from three experiments.

When considering the effect of the SBOFA loading from 0 to 8 phr, it was found that both the M_H_ and M_H_-M_L_ values passed through the maximum at 4 phr (Figure 8a). Generally, M_H_-M_L_ value could be indirectly used as an indication of the degree of crosslink density in the rubber vulcanizates [13]. The increased M_H_-M_L_ value when increasing the SBOFA content from 2 to 4 phr would be due to the increased crosslink level in the rubber. However, the M_H_-M_L_ value declined when further increasing SBOFA from 4 to 8 phr, which might be attributed to the excess amount of SBOFA loading (above 4 phr) significantly reducing the physical crosslinking and giving a greater plasticizing of the rubber matrix.

To confirm that the SBOFA-plasticized NBR with a SBOFA loading of 4 phr had the greatest crosslink density, the crosslinking densities from the swelling measurement of the NBR compound were plotted against the SBOFA loading [Figure 8b]. In a similar trend to the M_H_-M_L_ value, the crosslink density passed through a maximum at a SBOFA loading of 4 phr. The increased crosslink density with increasing SBOFA loading from 2 to 4 phr was contributed by the unsaturated content of SBOFA predominantly participating in the crosslinking reaction [10]. However, above 4 phr of SBOFA, the crosslink density tended to decrease despite the compound due to the excess amount of SBOFA excessively reducing the rubber–rubber interaction and increasing the free volume, thus allowing the solvent to more easily penetrate into the rubber matrix. This can lead to the observed higher swelling and lower calculated crosslink density.

The ATR-FTIR spectra of the NBR vulcanizates as presented in Figure 6 were used to compare the crosslink density of NBR vulcanizates when varying SBOFA loadings (0–8 phr). The intensity ratios of peaks at 1660–1640, 1538, and 806 cm^−1^ to the reference peak at 1443 cm^−1^ given as R_1_, R_2_, and R_3_ are depicted in Table 4. It was observed the NBR vulcanizate with 4 phr SBOFA showed the lowest R_1_, R_2_, and R_3_. This implies that this sample had the least level of unsaturated bond left. In addition, the occurrence of peroxide crosslinking was also indicated by the intensity ratio of the C–C stretching peak at 1140 and 1024 cm^−1^ to the reference peak at 1443 cm^−1^ represented as R_4_ [30]. After peroxide curing, when the ratio of these peaks is higher, there will be more C–C bonds, which implies the formation of crosslinking. From Table 4, it is obvious that the NBR vulcanizate without SBOFA showed the highest R_4_ (0.79) while amongst the SBOFA plasticized NBR vulcanizates, the one with the SBOFA 4 phr gave the highest R_4_ (0.76). Thus, it was concluded that SBOFA 4 phr gave the NBR vulcanizate the highest crosslink density. It is noted that R_1_, R_2_, and R_3_ showed the same order as the M_H_-M_L_ value and crosslink density (Figure 8), ranked as NBR with SBOFA at 0 phr > 4 phr > 2 phr > 6 phr > 8 phr.

Furthermore, Table 5 lists the cure characteristics in terms of the scorch (t_s2_) and cure times (t_90_) along with the cure rate index (CRI) values of the compounds with various SBOFA contents. It was observed that the presence of SBOFA at a loading of 6 phr or less did not obviously affect the scorch time (t_s2_) and cure time (t_90_). The slight difference may be attributed to the deviation of the measurement. However, when loading SBOFA at 8 phr, the NBR compound showed a longer scorch time (t_s2_) and cure time (t_90_). Regardless of SBOFA loading, all SBOFA plasticized NBR compounds showed comparable CRI and negligibly less than the unplasticized NBR compound. The retardation of vulcanization by SBOFA at a high loading may be attributed to the effect of its substantially unsaturated fatty acid contents [10,31]. Thus, it was concluded that the addition of an excess SBOFA interrupted the crosslinking reaction more than its participation in the vulcanization. This is also relevant to the reduced degree of crosslink density (i.e., M_H_-M_L_ and crosslink density), as shown in Figure 8.

### 3.5. Influence of the SBOFA Loading on the Mechanical Properties of NBR Vulcanizates

**Figure 9:**
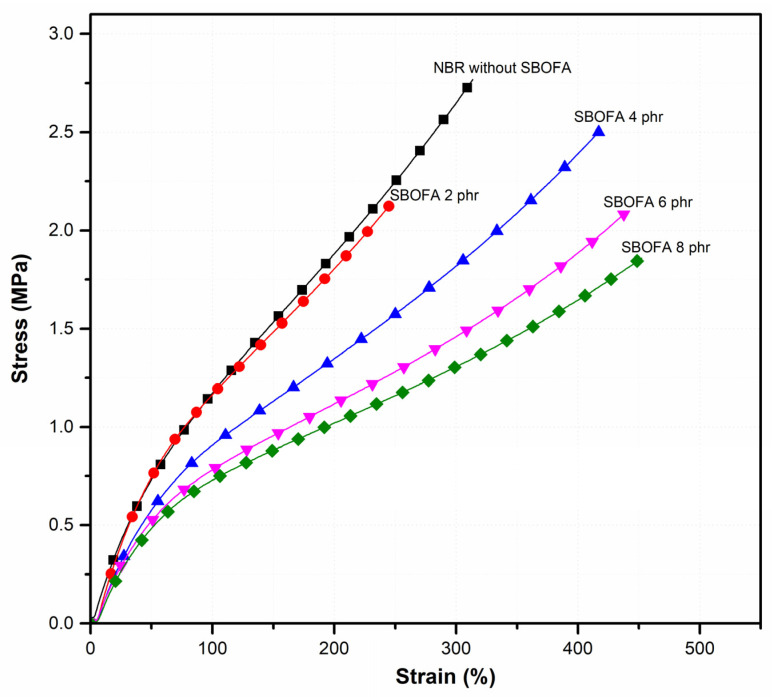
Stress–strain curves of the NBR vulcanizates with varying SBOFA loadings.

Figure 9 shows the stress–strain behavior of the NBR vulcanizates with varying SBOFA loadings. Table 6 summarizes the modulus at 100%, 200%, and 300% of the NBR vulcanizates. It was observed that when increasing the SBOFA loading, the 100%, 200%, and 300% modulus continuously decreased, as seen in Table 6. This could be attributed to the increased free volume and decreased physical entanglement with the presence of SBOFA molecules, thus facilitating the rubber chains to move upon applying the external force.

**Figure 10:**
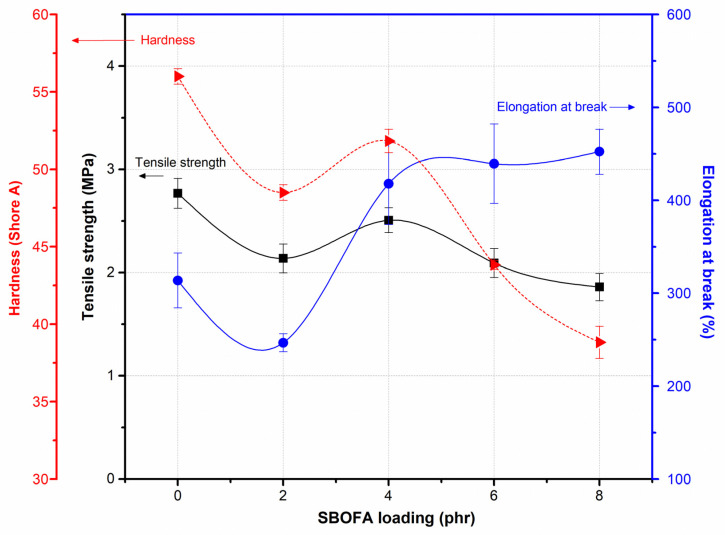
The tensile strength, elongation at break, and hardness of NBR vulcanizates with varying SBOFA loadings. Data of tensile properties and hardness are shown as the mean ± 1SD, derived from five and three experiments, respectively.

The effect of SBOFA loading on the tensile strength, elongation at break, and hardness of the NBR vulcanizates is illustrated in Figure 10. As seen, the tensile strength of the NBR vulcanizate changed from 2.77 ± 0.20 to 2.14 ± 0.14, 2.51 ± 0.29, 2.09 ± 0.14, and 1.86 ± 0.13 MPa when increasing the SBOFA loading from 0 to 2, 4, 6, and 8 phr, respectively. Even for the plasticized NBR vulcanizates the tensile strength seemed to pass through a maximum at the SBOFA loading of 4 phr; taking into account the measurement errors, there is a minimal difference between the results obtained when varying the SBOFA loadings. In addition, it can be concluded that within the range of SBOFA loadings studied here the presence of SBOFA had no significant effect on the tensile strength of NBR vulcanizates.

In contrast, the presence of SBOFA seemed to play an obvious role in the elongation at break and hardness. An increased SBOFA loading improved the elastic properties, as indicated by the increased elongation at break (Figure 9 and Figure 10). The highest elongation at break (452%, Figure 10) was found in the NBR loaded with 8 phr SBOFA. The change in the elongation at break was in the order of SBOFA loading at 8 phr > 6 phr > 4 phr > 0 phr > 2 phr, as shown in Figure 10. This can be explained by the increased movement of rubber molecules in the larger free volume between the entanglements of rubber molecules that is induced by SBOFA, resulting in the reduced crosslink density. This gives the opposite consequence to that of the crosslink density (M_H_-M_L_ and crosslink density) of the vulcanizates (Figure 8). The presence of SBOFA caused the vulcanizates to have a lower hardness than without SBOFA. The hardness tended to decrease with increasing the SBOFA loading except at the SBOFA 4 phr, at which its resultant vulcanizate showed the highest hardness. Possibly, it could be because amongst all SBOFA plasticized-NBR vulcanizates this one had the highest crosslink density.

**Figure 11:**
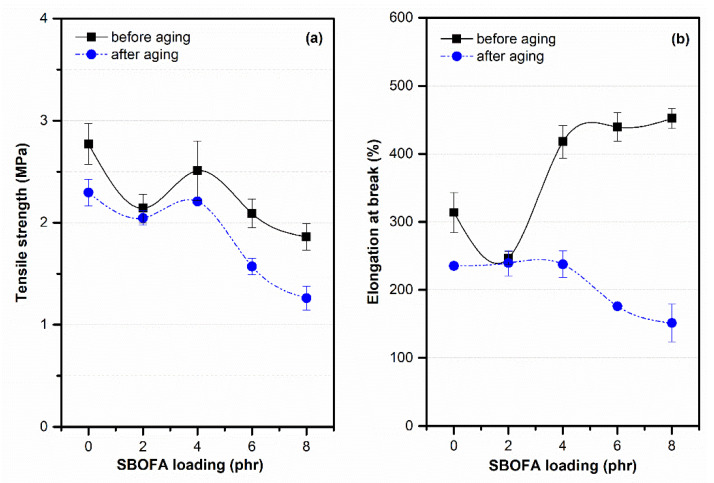
The (**a**) tensile strength and (**b**) elongation at break of NBR vulcanizates with varying SBOFA loadings before and after thermal aging at 100 °C for 48 h. Data are shown as the mean ± 1SD, derived from five experiments.

The aging resistance was evaluated in terms of the mechanical properties of each sample after thermal aging at 100 °C for 48 h, with the data summarized in Table 7. After thermal aging for 48 h, all the NBR vulcanizates both with and without SBOFA exhibited a lower tensile strength and a lower elongation at break than the unaged ones, as shown in Figure 11.

Moreover, the retention in the mechanical properties (i.e., tensile strength and elongation at break) after thermal aging at 100 °C for 48 h are given in Table 7. The retention in the tensile strength of the NBR vulcanizates increased with SBOFA loadings up to 4 phr and then significantly decreased with further increasing SBOFA loadings above 4 phr (Table 8). That a suitable SBOFA loading (2–4 phr) could improve the thermal stability of the NBR vulcanizates is conceivably due to the existence of natural antioxidants (i.e., tocopherols) in the unsaturated fatty acids of SBO that enhance the thermal stability of the NBR vulcanizate [32]. It may also be attributed to higher crosslink density which retarded the thermal degradation of the materials. Furthermore, the retention in elongation at break at 100 °C decreased with increasing SBOFA loadings (Table 7). This behavior was caused by both the level of crosslink formation and the main chain scissions at high temperatures, which then caused a reduced elongation of the polymer. The degradation action of rubber molecules by oxygen, especially in the butadiene segment, is activated by thermal aging [33]. Thermo-oxidative aging was also characterized by an aging coefficient (*A_f_*), as shown in Table 7. Comparable to the % retention, it was depicted that the presence of small SBOFA loading (2–4 phr) can enhance thermal resistance of the NBR vulcanizates and vice versa.

To further investigate these results, the functional groups of the NBR vulcanizates after thermal aging at 100 °C for 48 h were evaluated using ATR-FTIR analysis with the results summarized in Table 9. The absorption peak at 1024 and 1140 cm^−1^ (due to the C–C bond) were reduced, while the peaks at 1740 and 1710 cm^−1^ (C=O), and 3310 and 3450 cm^−1^ (–OH), increased after aging. This can be explained by the increase in some functional groups by thermo-oxidation in the aging process. In addition, the lower intensity ratio of the C–C bond peak to reference peak at 1443 cm^−1^, due to the generation of free radicals by thermal energy during aging, led to the formation of carbon radicals in the molecules [34]. Moreover, the changes in the intensity ratio of peaks at 3310 and 3450 cm^−1^ (–OH) to reference peak at 1443 cm^−1^ indicated the increased occurrence of hydroperoxide, carboxylic acid, and –OH after aging [35].

In contrast, the modulus at 100% and 200% together with hardness of all NBR vulcanizates slightly increased after thermal aging (Table 8). This may be due to the partial loss of the SBOFA or its volatilization during thermal aging, while it may also be attributed to the reduction in the unsaturated bond contents in the vulcanizates [36]. This led to the samples becoming more rigid (increased hardening) after thermal aging, and corresponds well to the main change in the C–H stretching vibration where a lower peak intensity ratio was found in all NBR vulcanizates after thermal aging. Furthermore, the reduction of C=C in the range of 1660–1640 cm^−1^ can indicate the increased crosslink density [35] of the aged NBR vulcanizates (Table 9). The reduced intensity ratio of the peak at 1660–1640 cm^−1^ to the reference peak at 1443 cm^−1^ is implied to result from chain scission and the subsequent formation of crosslinking via the double bond occurred during thermal aging [35]. The double bonds in NBR molecules can form with alkyl and/or alkoxy radicals under thermal aging. These radicals can be oxidized further to form –C=O and –OH and can merge with each other to form crosslink formation [35]. This led to the increase in crosslink density, thus resulting in slight enhancement in modulus (i.e., 100% and 200%) and hardness of the aged NBR sample (Table 8).

### 3.6. Influence of the SBOFA Loading Level on the Stress-Relaxation Behavior of NBR Vulcanizates

**Figure 12:**
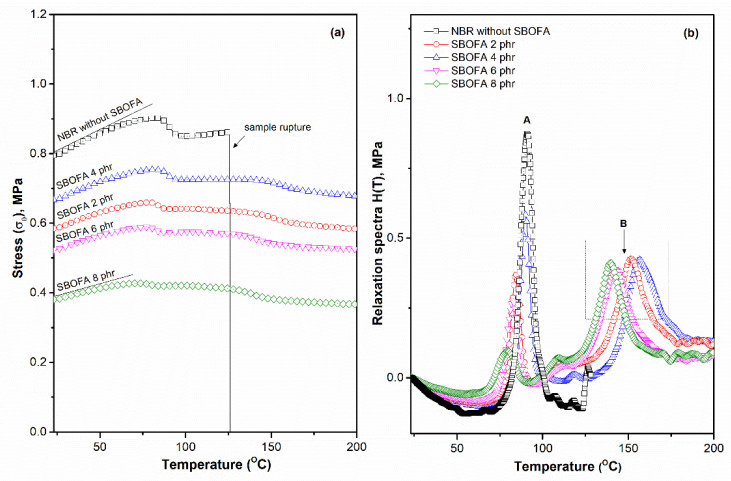
Representative (**a**) stress (σ_0_) and (**b**) relaxation spectra H(T) plots as function of temperature for NBR vulcanizates with varying SBOFA loadings.

Stress (σ_0_) and relaxation spectra H(T) as a function of the temperature of the NBR vulcanizates with varying SBOFA loadings (0–8 phr) are shown in Figure 12, which analyzed by temperature scanning stress relaxation (TSSR) measurement. The initial stress (σ_0_) clearly diminished in the presence of SBOFA, which is due to the plasticizing effects of the SBOFA [37] that increases the mobility of the polymer under the applied force. It is noteworthy that the σ_0_ could indicate the stiffness (ability to resisting deformation) of the materials [38]. The σ_0_ passed through a maximum value at a SBOFA loading of 4 phr, which was caused by the higher crosslink density, as mentioned earlier, resulting in an enhanced stiffness of the vulcanizates. Hence, the σ_0_ of the NBR vulcanizates showed a very similar trend to the hardness (Figure 10), as summarized in Table 10, which is related to the stiffness of the material.

Furthermore, at the early stage, the stresses slightly increased with temperature due to an entropy effect [18], similar to the level of crosslinking seen in Figure 12a. That the SBOFA loading at 4 phr increased the entropy effect was attributed to the unsaturated content of SBOFA, which participated in the crosslinking reaction and led to an increased stress in the material (Figure 12a), while the decreased slope of the stress in the temperature range 0–80 °C caused the stress relaxation of peak A (Figure 12b). This behavior is overshadowed by the entropy effect caused by the disruption of the weak physical interactions in rubber molecules. Furthermore, the stresses of all NBR vulcanizates slightly decreased and dropped down close to zero at temperatures above 150 °C (Figure 12a). This can be explained by the partial thermal splitting of peroxide crosslinking or the thermo-oxidative splitting of rubber molecules [18].

Figure 12b shows the relaxation spectra H(T) as a function of the temperature for NBR vulcanizates with varying SBOFA loadings. Two relaxation peaks were observed over the temperature range 23–220 °C. Peak A, in the temperature range 50–80 °C, might be correlated to the desorption of physical interactions [38]. Peak B, in the temperature range 110–190 °C, was attributed to chemical interactions caused by the cleavage of peroxide crosslinking and chain scission of the rubber molecules [38]. The maximum of peak A (Table 10) was slightly shifted to a lower temperature in the relaxation spectrum of NBR with a SBOFA loading above 4 phr, which is due to the dilution effect where a greater plasticization resulted in weak physical interactions [10] (a looser network of rubber molecules) with higher SBOFA loading levels. This led to the easy detachment of these interactions as the temperature increased.

**Figure 13:**
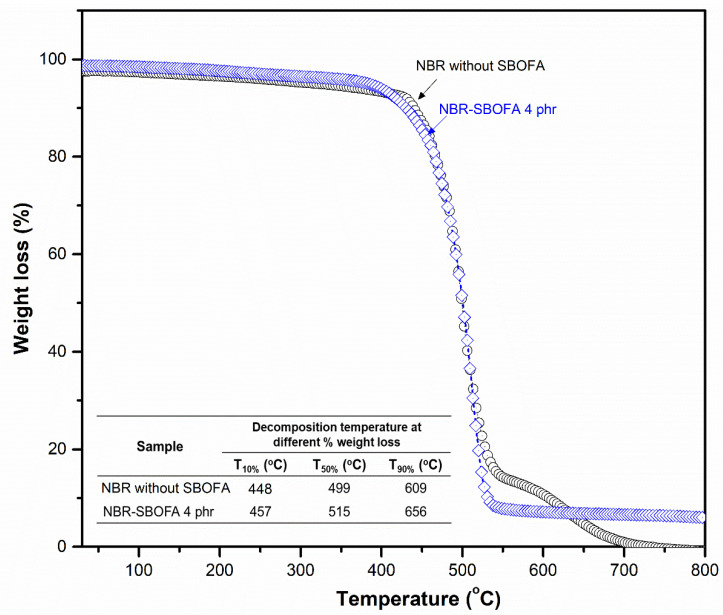
Representative TGA curves of the NBR vulcanizates with SBOFA at 0 and 4 phr.

Furthermore, it is clear that the height and area underneath the relaxation peak B in the temperature range of 110–190 °C [Figure 12b] increased with a decreasing SBOFA loading below 4 phr. The highest height of peak B was also observed in NBR with 4 phr of SBOFA, while it shifted forward to a higher temperature (about 157 °C). This reflects the better thermal stability of the NBR vulcanizate [39], which may be caused by the strong interaction between the rubber and SBOFA and the high level of peroxide crosslinking that led to the increased relaxation energy (Figure 12b). This result corresponded well with the initial stress (Figure 12a) and is ranked in the order of NBR with SBOFA at: 0 phr > 4 phr > 2 phr > 6 phr > 8 phr, which is consistent with the level of crosslink density (Figure 8 and Table 10) and correlates well to the optimal thermal stability of the NBR vulcanizates, as ascertained using TGA (Figure 13).

Figure 13 shows the TGA curves and the decomposition temperatures at different mass changes of the samples in relation to the initial mass (e.g., 10%, 50%, and 90%). An improved thermal stability of the NBR vulcanizate with 4 phr SBOFA is clearly seen compared to that with no SBOFA, as also evident from the higher temperature of T_10%_, T_50%_, and T_90%_. The higher thermal stability was possibly due to the interaction between the SBOFA and NBR [40]. Furthermore, it was observed in the temperature range of 500–800 °C the weight loss of the NBR with 4 phr SBOFA was gradually close to a straight line with the 6% of remaining weight, whereas that of the NBR without SBOFA further increased [40]. This may be due to the SBOFA molecules acting as a protective barrier, which deterred the release of volatile degradation products out from the sample and suppressed the thermal degradation [41]. This result is correlated well to the relaxation spectra H(T) which hence indicated thermal stability of the material, as shown in Figure 12b. As a result, it can be concluded that the thermal stability of the vulcanizate can be improved by the addition of a suitable SBOFA loading level, which was 4 phr in this case.

Moreover, it is noteworthy that the stress–temperature curves obtained from the TSSR measurement can be applied to estimate the degree of crosslink density from the initial slope (plateau region; Figure 12a) by applying a modified neo-Hookean equation [38], as shown in Equation (6):

(6) $$\sigma \;\left(mol/m^{3}\right)=v\cdot R\cdot T\;\left(\frac{\lambda }{1+\alpha \left(T-T_{0}\right)}-{\left(\frac{\lambda }{1+\alpha (T-T_{0)}}\right)}^{-2}\right)$$

where λ = *I*/*I_0_* is the extension ratio and α is the thermal expansion coefficient (2.4 × 10^−4^ K^−1^), and *σ* and *R* are the stress and the universal gas constant, respectively.

Table 10 summarizes the degree of the crosslink density of the NBR vulcanizates with varying SBOFA loadings based on the TSSR analysis. The crosslink densities of all the vulcanizates obtained from TSSR measurement showed the same trend as those determined by the swelling method (Figure 8). The highest crosslink density (about 211 mol/m^3^) was found in the NBR with 4 phr SBOFA loading followed by those with SBOFA at 2, 6, and 8 phr at about 169, 157, and 143 mol/m^3^, respectively (Table 10). As a result, it can be concluded that a 4 phr SBOFA loading is the optimal amount to enhance the initial stress and area underneath the relaxation peaks. Thus, these results appear to be relevant to the M_H_-M_L_ value, level of crosslink density (Figure 8), hardness (Table 6), and the initial stress (σ_0_) (Table 10), together with thermal stability of the vulcanizates (Figure 13).

### 3.7. Comparison of the Effect of SBOFA and DOP on the Properties of NBR Vulcanizates

For a long time, DOP has been commercially used as a processing aid for NBR. It is an oily liquid that does not react chemically with rubber, and so can be classified as a physical plasticizer. Due to the requirement to avoid any toxic effect, the aim of this study was to propose an alternative processing aid derived from a natural seed crop for NBR, since it was reported in the previous section that the use of SBOFA could significantly reduce the Mooney viscosity of NBR compounds. The higher the SBOFA loading level, the lower the viscosity. However, increasing SBOFA amounts above 4 phr seemed to reduce all the mechanical properties. Therefore, the SBOFA loading used in this comparative study was 2–4 phr. To compare the performance of SBOFA with that of the commercial DOP, the properties of NBRs loaded with SBOFA at 2 and 4 phr were discussed with respect to those loaded with DOP at an equivalent level.

**Figure 14:**
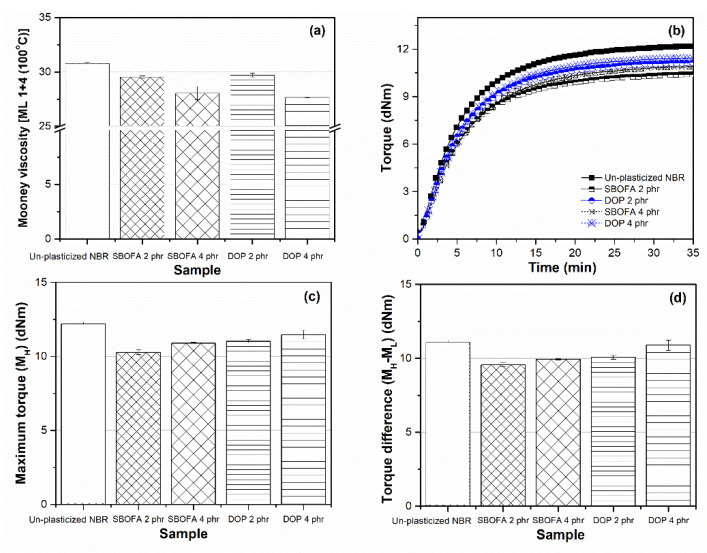
The (**a**) Mooney viscosity; (**b**) cure characteristic; (**c**) maximum torque (M_H_), and (**d**) torque difference (M_H_-M_L_) of unplasticized NBR and NBR vulcanizates with 2 and 4 phr of SBO and DOP. Data are shown as the mean ± 1SD, derived from three experiments.

Figure 14a shows the Mooney viscosity of rubber compounds with SBOFA and DOP at 2 and 4 phr in comparison with that of rubber compounds without any processing aids as a reference. Regardless of type and loading of processing aid, all plasticized rubber compounds showed lower Mooney viscosity than the unplasticized one. At either loading level, the SBOFA-plasticized compounds showed a comparable Mooney viscosity with the DOP-plasticized ones. From Figure 14, it was observed that the M_H_ and M_H_-M_L_ values of the SBOFA-plasticized NBRs at 2 and 4 phr were not significantly different from those of the DOP-plasticized NBRs. Table 11 summarizes the cure parameters of the NBR vulcanizates with SBOFA and DOP at 2 and 4 phr. The use of SBOFA gave the rubber compound a slightly longer t_s2_ and t_90_ than the use of DOP, since the unsaturated fatty acid content in the SBOFA led to the retardation of the peroxide vulcanization [42]. However, the CRI of the SBOFA- and DOP-plasticized rubbers were not significantly different.

**Figure 15:**
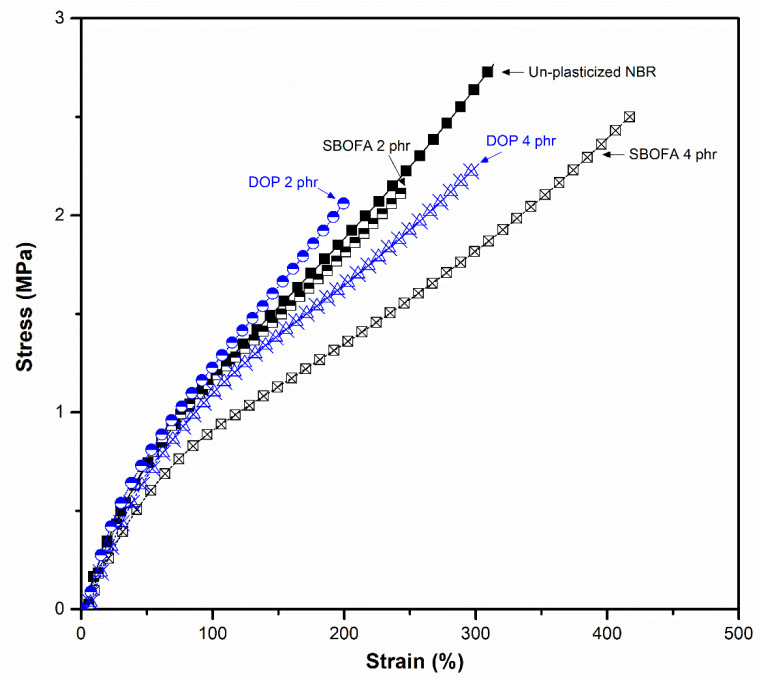
Representative stress–strain curves of unplasticized NBR and NBR vulcanizates with 2 and 4 phr loadings of SBO and DOP.

Comparison of the effect of the SBOFA and DOP at 2 and 4 phr on the tensile properties of the obtained rubber vulcanizates is shown in Figure 15 and Table 12. The tensile strength of the SBOFA-plasticized NBRs were nearly comparable with that of the DOP-plasticized NBRs at each given loading. Nevertheless, it was found that the SBOFA-plasticized NBR vulcanizates at any given loading showed a higher elongation at break than the DOP-plasticized NBR ones. The SBOFA-plasticized NBR at 4 phr could be extended up to the 400%. This behavior may be due to the better plasticization of SBOFA compared to DOP [43]. Regardless of the type of processing aid, the results showed that the modulus at 100% and 200% of the plasticized-NBR gradually decreased with increasing processing aid loading. This was due to the dilution of the plasticization [10]. There was no noteworthy difference in modulus observed when using different processing aids. In addition, the SBOFA-plasticized NBRs showed a slightly higher hardness than the DOP-plasticized NBRs did at each given loading, as seen in Table 12.

Thus, it can be concluded that SBOFA is a good alternative processing aid for NBR. This is because the use of SBOFA will reduce the toxicity of the final product for the rubber industry but provide the NBR compounds with the comparable viscosity and performance in terms of tensile strength and hardness to those containing DOP. An additional benefit when using SBOFA as a processing aid is offering the NBR vulcanizates a significantly higher elongation at break than with DOP.

## 4. Conclusions

The results of this study show that SBOFA derived from a natural seed crop is a potential alternative processing aid for NBR in the rubber industry. Comparative to the synthetic processing aids, such as DOP, SBOFA is an environmentally friendly processing aid derived from a renewable resource and can be simply obtained from a hydrolysis of SBO with NaOH. The SBOFA showed a comparable plasticizing efficiency with the DOP. Although the SBOFA-plasticized NBR compounds needed a slightly longer curing time than the DOP-plasticized NBR compounds, the SBOFA-plasticized NBR vulcanizate showed a similar tensile strength and hardness to the DOP-plasticized NBR vulcanizates. For any application in which high elongation is required, the use of SBOFA as a processing aid was superior to the use of DOP. This was due to the SBOFA obviously imparting more chain flexibility to the rubber chain than DOP.

## Figures and Tables

**Table 1:** Characteristic and sources of the chemicals used.

Chemicals	Sources
Soybean oil (SBO)	Lotus’s, Ek-Chai Distribution System Co., Ltd., Khet Buengkoom, Bangkok, Thailand
Sodium hydroxide (NaOH)	Fisher Scientific, Loughborough, UK
Sodium chloride (NaCl)	Merck KGaA, Darmstadt, Germany
Hydrochloric acid (HCl)	Merck KGaA, Darmstadt, Germany
Acrylonitrile-butadiene rubber (NBR) (1052, acrylonitrile content 33%)	Nantex Industry Co., Ltd., Zhenjiang, Jiangsu, China
Dioctyl phthalate (DOP)	Hansen & Rosenthal Chemical Co., Ltd., Hamburg, Germany
Dicumyl peroxide (DCP)	A.F. Supercell Co., Ltd., Khlong Toei, Bangkok, Thailand

**Table 2:** Formulations of rubber compounds.

Ingredients	Processing Aid Loading (Parts per Hundred Rubber; phr)						
	0	2	4	6	8	2	4
NBR (ACN 33%)	100.0	100.0	100.0	100.0	100.0	100.0	100.0
DCP	2.0	2.0	2.0	2.0	2.0	2.0	2.0
SBOFA	-	2.0	4.0	6.0	8.0	-	-
DOP	-	-	-	-	-	2.0	4.0

**Table 3:** Potential peak assignments of the ATR-FTIR spectra from Figure 1, Figure 5 and Figure 6.

Wave Number cm^−1^	Assignment
3500–3300	O–H stretching vibration of carboxyl and hydroxyl groups
3008	–C=CH stretching
2926	C–H of CH_3_ stretching
2919	CH_2_ stretching vibration
2854	C–H of CH_2_ stretching
2848	CH_2_ stretching vibration
2236	–C≡N stretching vibrations in the acrylonitrile segments
1743–1740	C=O stretching vibration of ester
1710	C=O stretching vibration of carboxylic acid
1660–1640	C=C stretching vibration
1578	Carboxylate ion (COO–) stretching vibration
1538	C=C symmetric stretching vibration of double bond
1464	C–H of CH_2_ stretching
1443	C–H bending vibration in –CH_2_
1262	Stretching vibrations of C–O in C–OH groups
1160	–CH in plane
1024, 1140	C–C stretching vibration
966	C–H out-of-plane bending vibration of *trans*-1,4 –HC=CH–
919	Out of plane –CH=CH_2_ deformations
806	Vibrations of residual –C=C– bonds
722	–HC=CH– bending (out of plane)

**Table 4:** Peak ratios of the ATR-FTIR spectra representing the level of double bonds (–C=C–) and new C–C linkage in the peroxide-vulcanized NBR.

Sample	Level of –C=C– Bonds			Level of C–C Linkages
	Peak Ratio *R*_1_ *= A*_1660−1640_/*A*_1443_	Peak Ratio *R*_2_ = *A*_1538_/*A*_1443_	Peak Ratio *R*_3_ =*A*_806_/*A*_1443_	Peak Ratio *R*_4_ =*A*_1140+1024_/*A*_1443_
NBR without SBOFA	0.37	0.13	0.13	0.79
NBR-SBOFA 2 phr	0.49	0.21	0.18	0.71
NBR-SBOFA 4 phr	0.44	0.19	0.17	0.76
NBR-SBOFA 6 phr	0.49	0.22	0.25	0.69
NBR-SBOFA 8 phr	0.56	0.22	0.34	0.48

*R*_1_, *R*_2_, *R*_3_, and *R*_4_ parameters are peak ratios calculated from the absorption peak of NBR vulcanizates at 1660–1640, 1538, 806, and 1140 + 1024 cm^−1^ to the reference peak at 1443 cm^−1^.

**Table 5:** Cure parameters of NBR compounds with varying SBOFA loadings.

Cure Parameters	SBOFA Content (phr)				
	0	2	4	6	8
Scorch time (t_s2_) (min)	2.06 ± 0.05	2.45 ± 0.05	2.52 ± 0.04	2.63 ± 0.06	3.87 ± 0.09
Cure time (t_90_) (min)	15.01 ± 0.06	16.05 ± 0.08	16.08 ± 0.13	16.48 ± 0.18	17.21 ± 0.2
Cure rate index (CRI) (min^−1^)	7.72 ± 0.04	7.35 ± 0.02	7.37 ± 0.05	7.22 ± 0.06	7.5 ± 0.06
Crosslink density (mol/m^3^)	226.87 ± 3.10	156.45 ± 1.79	198.21 ± 3.43	147.32 ± 2.83	138.08 ± 2.42

Data are shown as the mean ± 1 SD, derived from three experiments.

**Table 6:** Modulus of the NBR with varying SBOFA loadings.

Mechanical Properties	SBOFA Loading (phr)				
	0	2	4	6	8
100% modulus (MPa)	1.19 ± 0.02	1.17 ± 0.02	0.91 ± 0.02	0.77 ± 0.05	0.70 ± 0.02
200% Modulus (MPa)	1.78 ± 0.03	1.70 ± 0.06	1.37 ± 0.04	1.10 ± 0.09	1.02 ± 0.02
300% Modulus (MPa)	-	-	1.86 ± 0.06	1.44 ± 0.14	1.33 ± 0.02

Data of modulus are shown as the mean ± 1SD, derived from five experiments.

**Table 7:** Retention in mechanical properties (tensile strength and elongation at break) and aging coefficient (*A_f_*) of the NBR vulcanizates with varying SBOFA loadings.

Sample	Retention in Tensile Strength (%)	Retention in Elongation at Break (%)	Aging Coefficient (*A_f_*)
NBR without SBOFA	82.86	75.01	0.62
SBOFA 2 phr	94.07	97.06	0.91
SBOFA 4 phr	94.37	57.59	0.54
SBOFA 6 phr	75.17	40.04	0.30
SBOFA 8 phr	67.80	33.47	0.23

**Table 8:** Mechanical properties of the aged NBR vulcanizates with varying SBOFA loadings.

Mechanical Properties (after Aging at 100 °C)	Soybean Oil Fatty Acid Content (phr)				
	0	2	4	6	8
100% modulus (MPa)	1.27 ± 0.09	1.19 ± 0.06	1.22 ± 0.12	1.06 ± 0.09	0.93 ± 0.14
200% Modulus (MPa)	1.99 ± 0.13	1.73 ± 0.08	1.89 ± 0.16	-	-
Hardness (Shore A)	58.0 ± 0.5	49.7 ± 1.0	54.5 ± 0.4	46.9 ± 0.4	41.1 ± 0.8

Data of tensile properties and hardness are shown as the mean ± 1SD, derived from five and three experiments, respectively.

**Table 9:** Peak ratios of the ATR-FTIR spectra that represent the C=C, C–C bond, C=O, and COOH at (1660–1640), (1140+1024), (3310 + 3450), and (1740 + 1710) cm^−1^/1443 cm^−1^ of the unaged and aged NBR vulcanizates.

Sample	Concentration of the Correlative Functional Groups (%)							
	(1660–1640 cm^−1^)/1443 cm^−1^		(1140 + 1024) cm^−1^/1443 cm^−1^		(1740 + 1710) cm^−1^/1443 cm^−1^		(3310 + 3450) cm^−1^/1443 cm^−1^	
	Unaged	Aged	Unaged	Aged	Unaged	Aged	Unaged	Aged
NBR without SBOFA	0.37	0.28	0.79	0.42	0.11	0.33	0.11	0.23
SBOFA 2 phr	0.49	0.28	0.71	0.69	0.15	0.35	0.17	0.31
SBOFA 4 phr	0.44	0.30	0.76	0.57	0.24	0.62	0.18	0.33
SBOFA 6 phr	0.49	0.25	0.69	0.48	0.25	0.68	0.19	0.35
SBOFA 8 phr	0.56	0.25	0.48	0.56	0.34	0.69	0.22	0.38

**Table 10:** Initial stress (σ_0_), peak areas in relaxation spectra over the temperature range 50–80 °C (Figure 11b), and the crosslink density based on the TSSR analysis for NBR vulcanizates with varying SBOFA loadings.

Rubber Sample	Initial Stress (σ_0_) (MPa)	Peak Area (MPa*K) (Temperature Range 50–80 °C)	Crosslink Density by TSSR Measurement (mol/m^3^)
NBR without SBOFA	0.79	4.27	237
SBOFA 2 phr	0.58	1.77	169
SBOFA 4 phr	0.67	2.34	211
SBOFA 6 phr	0.52	1.31	157
SBOFA 8 phr	0.38	1.29	143

**Table 11:** Cure parameters and crosslink density of unplasticized NBR and NBR vulcanizates with 2 and 4 phr loadings of SBO and DOP.

Cure Parameters	Unplasticized NBR	SBOFA 2 phr	SBOFA 4 phr	DOP 2 phr	DOP 4 phr
Scorch times, t_s2_ (min)	2.06 ± 0.05	1.99 ± 0.08	2.46 ± 0.01	2.05 ± 0.06	2.07 ± 0.08
Cure time, t_90_ (min)	15.01 ± 0.06	14.85 ± 0.10	16.05 ± 0.05	15.09 ± 0.08	14.94 ± 0.70
Cure rate index, CRI (min^−1^)	7.72 ± 0.04	7.77 ± 0.01	7.36 ± 0.02	7.67 ± 0.01	7.78 ± 0.36
Crosslink density (mol/m^3^)	226.87 ± 3.10	159.84 ± 1.54	203.42 ± 2.51	144.75 ± 0.51	198.65 ± 1.72

Data are shown as the mean ± 1SD, derived from three experiments.

**Table 12:** Mechanical properties and hardness of unplasticized NBR and NBR vulcanizates with 2 and 4 phr loadings of SBO and DOP.

Properties/Sample	Unplasticized NBR	SBOFA 2 phr	SBOFA 4 phr	DOP 2 phr	DOP 4 phr
100% modulus (MPa)	1.19 ± 0.02	1.17 ± 0.02	0.91 ± 0.02	1.23 ± 0.02	1.09 ± 0.04
200% modulus (MPa)	1.78 ± 0.03	1.70 ± 0.06	1.37 ± 0.04	2.06 ± 0.04	1.64 ± 0.03
300% modulus (MPa)	-	-	1.86 ± 0.06	-	-
Tensile strength (MPa)	2.77 ± 0.20	2.14 ± 0.14	2.51 ± 0.29	2.08 ± 0.14	2.24 ± 0.43
Elongation at break (%)	314 ± 29	247 ± 9	418 ± 43	202 ± 8	299 ± 11
Hardness (Shore A)	56.0 ± 0.5	48.5 ± 0.5	51.8 ± 0.8	42.1 ± 0.6	47.2 ± 0.2

Data of tensile properties and hardness are shown as the mean ± 1SD, derived from five and three experiments, respectively.
